# Correction: Preparation of a novel curcumin nanoemulsion by ultrasonication and its comparative effects in wound healing and the treatment of inflammation

**DOI:** 10.1039/d0ra90079f

**Published:** 2020-07-22

**Authors:** Niyaz Ahmad, Rizwan Ahmad, Ali Al-Qudaihi, Salman Edrees Alaseel, Ibrahim Zuhair Fita, Mohammed Saifuddin Khalid, Faheem Hyder Pottoo

**Affiliations:** Department of Pharmaceutics, College of Clinical Pharmacy, Imam Abdulrahman Bin Faisal University P. O. Box 1982 Dammam Kingdom of Saudi Arabia-31441 nanhussain@iau.edu.sa niyazpharma@gmail.com +966 13 333 0290 +966 13 333 5541, +966 53 120 3626; Department of Pharmaceutical Chemistry, College of Clinical Pharmacy, Imam Abdulrahman Bin Faisal University Dammam Kingdom of Saudi Arabia; Department of Natural Products and Alternative Medicine, College of Clinical Pharmacy, Imam Abdulrahman Bin Faisal University Dammam Kingdom of Saudi Arabia; Department of Pharmacology, College of Clinical Pharmacy, Imam Abdulrahman Bin Faisal University Dammam Kingdom of Saudi Arabia

## Abstract

Correction for ‘Preparation of a novel curcumin nanoemulsion by ultrasonication and its comparative effects in wound healing and the treatment of inflammation’ by Niyaz Ahmad *et al.*, *RSC Adv.*, 2019, **9**, 20192–20206, DOI: 10.1039/C9RA03102B.

The authors regret errors in Fig. 7 in the original article. The corrected Fig. 7 is shown below where the panels at 8 and 12 days for fusidic acid and 16 and 20 days for Cur-NE have been replaced.

**Fig. 7:**
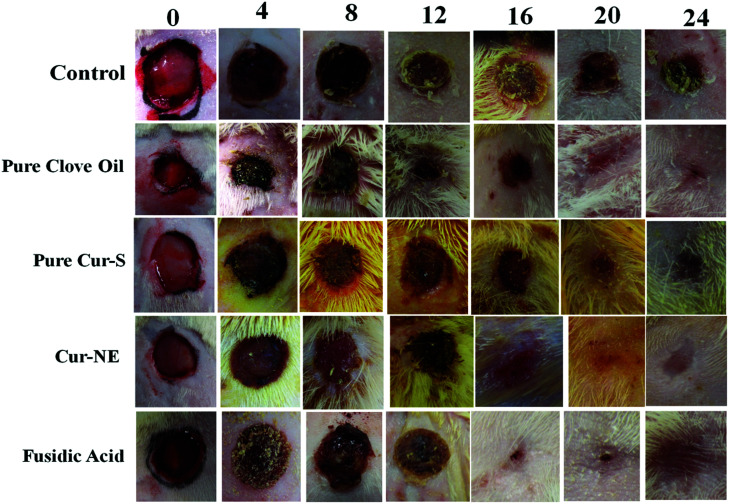
Wound healing effects of optimized nanoemulsion without Cur loaded; pure clove oil; pure Cur-S; optimized nanoemulsion and marketed preparation of antibiotic fusidic acid (Fusidin; positive control) in comparison with the control after 0, 4, 8, 12, 16, 20 and 24 days of inducing wound healing.

The Royal Society of Chemistry apologises for these errors and any consequent inconvenience to authors and readers.

## Supplementary Material

